# Gastroretentive Technologies in Tandem with Controlled-Release Strategies: A Potent Answer to Oral Drug Bioavailability and Patient Compliance Implications

**DOI:** 10.3390/pharmaceutics13101591

**Published:** 2021-09-30

**Authors:** Napoleon-Nikolaos Vrettos, Clive J. Roberts, Zheying Zhu

**Affiliations:** School of Pharmacy, University of Nottingham, Nottingham NG7 2RD, UK; napoleon.vrettos@nottingham.ac.uk (N.-N.V.); clive.roberts@nottingham.ac.uk (C.J.R.)

**Keywords:** stomach, absorption window, gastric retention, gastroretentive drug delivery systems, controlled release, patient compliance

## Abstract

There have been many efforts to improve oral drug bioavailability and therapeutic efficacy and patient compliance. A variety of controlled-release oral delivery systems have been developed to meet these needs. Gastroretentive drug delivery technologies have the potential to achieve retention of the dosage form in the upper gastrointestinal tract (GIT) that can be sufficient to ensure complete solubilisation of the drugs in the stomach fluids, followed by subsequent absorption in the stomach or proximal small intestine. This can be beneficial for drugs that have an “absorption window” or are absorbed to a different extent in various segments of the GIT. Therefore, gastroretentive technologies in tandem with controlled-release strategies could enhance both the therapeutic efficacy of many drugs and improve patient compliance through a reduction in dosing frequency. The paper reviews different gastroretentive drug delivery technologies and controlled-release strategies that can be combined and summarises examples of formulations currently in clinical development and commercially available gastroretentive controlled-release products. The different parameters that need to be considered and monitored during formulation development for these pharmaceutical applications are highlighted.

## 1. Introduction

Oral drug delivery systems are the most popular dosage forms for drug administration [1,2]. Many reasons contribute to their dominance, including high patient compliance, easy storage and transportation, cost-effectiveness and that no specialised medical personnel are required to administer. However, poor bioavailability can be an issue for many orally delivered drugs with pH-dependent solubility or stability or a narrow window of absorption. Such properties need to be considered during formulation development since they could cause incomplete drug absorption when the dosage form is transferred towards the lower part of the gastrointestinal tract (GIT) [3,4,5].

Gastroretentive drug delivery systems (GRDDS) were developed as a new approach for the oral controlled-release delivery of many drugs [6,7,8,9]. These systems can be retained in the stomach long enough to completely release the active drug from a formulation in the gastric fluids. Their application comes with several advantages, including improved absorption of drugs and reduced fluctuation in drug blood levels that lead to increased therapeutic efficacy and minimised adverse effects, as well as their potential to enable stomach-specific activity. These systems can ensure the controlled delivery of drugs for an extended period at the desired rate and absorption site [5,10,11,12]. Suitable candidates for GRDDS include drugs with poor absorption in the distal GIT, a narrow absorption window in the proximal small intestine, stability and/or solubility issues at alkaline intestinal pH, a short half-life and/or a local activity at the upper part of the intestine for the eradication of *Helicobacter pylori* [9,13,14,15,16,17,18,19,20,21].

GRDDS can either be controlled-release systems intrinsically or coupled with such technologies to ensure a controlled release of drugs. Controlled-release applications promote enhanced patient compliance to the treatment, due to less frequent administration, and minimised adverse effects, thereby promoting therapeutic efficacy [22,23,24,25,26,27].

Several strategies have been developed for gastric retention purposes. These include floating systems [13,14,16], high-density systems, bio/mucoadhesive [19,20,28,29,30,31], expandable [15], superporous hydrogels [32,33] and magnetic systems [34,35,36].

Different formulation-related factors can affect the quality and performance of the gastroretentive dosage form in terms of both gastric retention and controlled drug release. These include polymer types (non-ionic, cationic and anionic polymers), polymer composition in dosage form, viscosity grade, molecular weight of the polymer(s) that are used in the formulation, as well as drug solubility [16]. The size and density of gastroretentive systems can also be very important. Depending on the type of gastroretentive technology, it can either ensure a controlled release of active pharmaceutical ingredients (APIs) alongside gastric retention or it can be combined with a controlled-release strategy to ensure the release of drugs over the desired period.

This review examines the different gastroretentive drug delivery technologies available in the market and those reported in the literature, as well as their potential to ensure a controlled release of APIs either as standalone systems or coupled with controlled-release strategies. The potential and future of these combinatory applications are also discussed.

## 2. The Stomach

**Figure 1:**
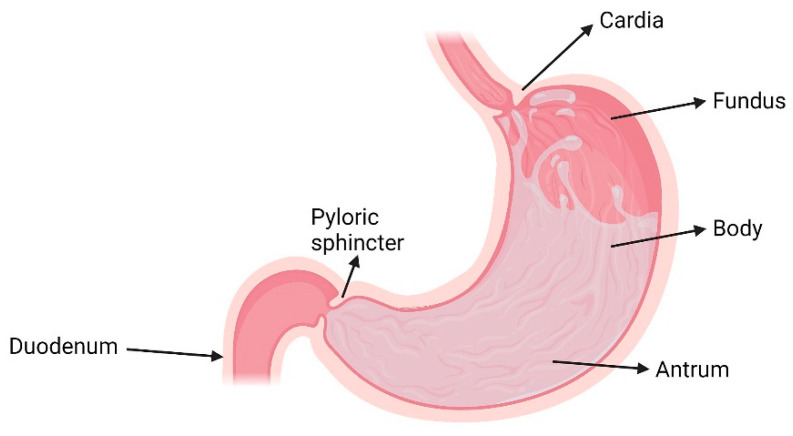
The human stomach. The figure was created using BioRender (www.biorender.com) (Accessed 2 August 2021).

The stomach (Figure 1) is a hollow, muscular organ located in the left upper quadrant of the peritoneal cavity [37]. Its role is to mix the orally ingested contents coming to the proximal stomach via the oesophagus, crossing the gastro-oesophageal junction (“cardia”), to form a chyme [38]. The distal stomach is connected to the duodenum and, the passage from the stomach to the duodenum is controlled by a muscular ring, i.e., the ‘pyloric sphincter’ or ‘pylorus’ [39]. The stomach is anatomically divided into three distinct regions: (i) the “fundus”, situated to the upper left of the cardia and is the proximal part of the stomach; (ii) the “body”, which lies between the fundus and the distal stomach (“antrum”) and is the biggest part of the stomach and (iii) the “antrum”, which constitutes the distal part of the stomach, ranging from the incisura angularis to the pylorus [38,40]. The body acts as a reservoir for undigested food materials, while the antrum acts as a pump aiming at assisting in gastric emptying of food particles through the pyloric sphincter via propelling actions. The antrum is the main mixing compartment of the stomach [41].

Gastric emptying occurs during both fasted and fed states. In the fasted state, the mobility of the stomach is controlled by the migrating myoelectric complex (MMC), which is a series of events occurring in a cyclic manner that lasts for about 2–3 h [42,43]. The MMC consists of four phases. Phase I includes rare contraction waves and lasts for about 30–60 min. Phase II is comprised of contractions of increasing intensity and frequency as time passes. This phase lasts for 20–40 min. Phase III lasts for 10–20 min and is characterised by intense, regular contractions that push the undigested materials through the pyloric sphincter and into the small intestine. For this reason, Phase III is also termed “housekeeper wave”. Finally, Phase IV includes a short transitional phase from Phase III to Phase I and lasts about 0–5 min [9]. This cycle is disrupted during the fed state, where the gastric emptying rate is lower. The motor activity is induced 5–10 min after ingestion and usually lasts 2–6 h, depending on the amount and caloric content of food [9].

As per Wickham et al., “Food in the stomach is usually a masticated mixture of protein, fat, carbohydrate, indigestible components, micronutrients, non-nutrient phytochemicals, microbiota, and water to which has been added a variable amount of saliva containing enzymes, salts, and bacteria. As such, the gastric food bolus is inhomogeneous at many different levels and retains, at least to some extent, the original structures of the foods consumed” [44]. Without the presence of food, the pH of the stomach is strongly acidic (pH~2.0) due to the presence of residual gastric secretions in the lowest part of the stomach the volume of which can be usually up to 50 mL. When food becomes increasingly present in the stomach, while the stomach wall pH may remain acidic, there is a rise in the stomach bulk pH towards that of the food mixture due to its high buffering capacity. The presence of food generates a rapid transition of gastric contractions while gastric emptying begins, with its rate varying, depending on meal properties, such as size, composition, viscosity, temperature, and osmolarity [44].

The strength of the contractions rises from the fundus to the antrum which is the mixing and pumping component of the stomach. Due to the higher shear and mixing conditions in the antrum, the contents there tend to be more homogeneous than in the main body; however, due to differences in the nature of chewed food items, a complete homogeneity of the antrum content is unlikely, unless food is in a liquid form [44].

When gastroretentive drug delivery systems are administered in the fasted state, the MMC may be in any of its phases. This can significantly affect the gastric retention time (GRT) of the systems [9].

## 3. Factors Affecting the Efficacy of GRDDS

Different factors can affect the performance of GRDDS. These can be categorised into formulation-related factors, physiological factors, and patient-related factors.

### 3.1. Formulation-Related Factors

It is important to understand the critical attributes of the different types of GRDDS that can affect their performance. In formulation design, a careful choice of excipients and polymers is necessary to ensure effective gastric retention. Swelling polymers which reduce dosage form density, such as hydroxypropylmethylcellulose (HPMC) and polyethylene oxide (PEO), are effective in achieving prolonged buoyancy and enabling floatation [45,46]. Polymers with strong mucoadhesive properties, such as Carbopol^®^ and chitosan, can assist gastric retention of the dosage form on the stomach wall [47,48]. In addition, other excipients that can also affect the system performance need to be considered, such as gas-generating agents for effervescent floating dosage forms and materials with swelling properties for superporous hydrogel systems.

The size and shape of the dosage form are also important parameters that can affect the performance of GRDDS. Gastric emptying occurs via the pyloric sphincter which has been reported to have a diameter of (12.8 ± 7) mm [46]. It is generally accepted that a dosage form size larger than 15 mm is required for effective gastric retention [9]. The ability of single-unit dosage forms to avoid premature evacuation from the stomach can be variable and this constitutes a major drawback for these systems. More usefully, multiple-unit systems, such as microparticle-based formulations, tend to be evacuated in a linear manner from the stomach or as a bolus at the end of digestion, thus providing a more reliable gastric retention and drug release behaviour [49]. Considering dosage form shape, tetrahedron- and ring-shaped dosage forms tend to have longer GRTs than others [10,50].

The density of the formulation is a critical factor for low- and high-density systems. The density of low-density systems should be lower than the estimated 1.004 g/cm^3^ value of gastric fluids, to allow for their floatation in the stomach [46,51,52]. However, the buoyancy duration of low-density dosage forms can also be dependent on the rate of hydration of the formulation [53]. For high-density systems, density should be higher than that of the gastric contents to ensure an effective sinking in the bottom of the stomach and resistance to the peristaltic movements; 2.5 g/cm^3^ is considered crucial for a prolonged GRT of these formulations [54].

### 3.2. Physiological Factors

Many studies have highlighted the importance of physiological factors in promoting or impeding the efficacy of GRDDS through affecting their gastric retention capability, including the amount, nature and caloric content of food, frequency of food intake, posture, sleep and physical activity [5,10,15,55,56,57]. During the fasted state, stomach mobility is controlled by the MMC which occurs cyclically. If the timing of formulation administration coincides with that of the MMC, then the GRT of the dosage form can be very low [58]. However, in the presence of food, the MMC and the housekeeper waves are disrupted, thus leading to a potentially prolonged GRT [10,41,59]. Furthermore, the administration of successive meals can increase gastric retention by 6-7 h, compared to a single intake of a meal. An increase in the caloric density of a meal can significantly prolong the GRT of dosage forms, whether it is protein-rich or fat-rich [56,58]. The nature of calories seems to have a minor effect on GRT [60]. In addition to the above, food viscosity is considered to have a positive effect on GRT [61,62]. Posture can affect different GRDDS in different ways. An upright position favours floatation of low-density systems, however, it impedes gastric retention capability of high-density systems, because they remain continuously in the lower part of the stomach and peristaltic contractions can result in a faster gastric emptying rate [5,10]. In contrast, in a supine position, the GRT of the non-floating systems is prolonged, compared to that of the floating systems [63,64]. Finally, the concomitant administration of drugs that affect the mobility of the gastrointestinal tract, such as anticholinergics, opiates and prokinetic agents, can affect the GRT [58].

### 3.3. Patient-Related Factors

Patient-related factors on the efficacy of GRDDS include age, gender, health conditions and emotional state. People aged over 65 tend to demonstrate longer GRTs for the dosage forms [65]. Wang et al. demonstrated that gender could have a significant effect on gastric emptying time and luminal pH with females demonstrating slower gastric emptying rates than males [66], possibly related to the menstrual cycle which has demonstrated an influence on the gastrointestinal (GI) transit. Wald et al. reported the prolongation of GI transit time during the luteal phase of the menstrual cycle, compared to the follicular phase which could mean that the transit is retarded with increasing progesterone levels [67]. Additionally, different health conditions can have varying effects on the GRT of dosage forms. Patients with Parkinson’s disease tend to have longer GRTs that may be accompanied by constipation [68]. In patients with diabetes mellitus, the gastric emptying rate seems to be significantly prolonged, compared to non-diabetic patients [69]. Finally, the emotional state of a patient seems to affect gastric emptying time. Patients with depression have demonstrated slower gastric emptying rates, while increased gastric emptying rates have been recorded in patients suffering from anxiety conditions [5,55,59].

## 4. Gastroretentive Drug Delivery Technologies and Their Controlled-Release Applications

There are a few different gastroretentive drug delivery approaches. These include floating, high-density, mucoadhesive/bioadhesive, expandable, superporous hydrogel and magnetic systems, as well as combinatory approaches of these technologies. Products based on gastroretentive technologies that are either under clinical trials or commercially available are summarised in Table 1.

### 4.1. Floating Systems

**Figure 2:**
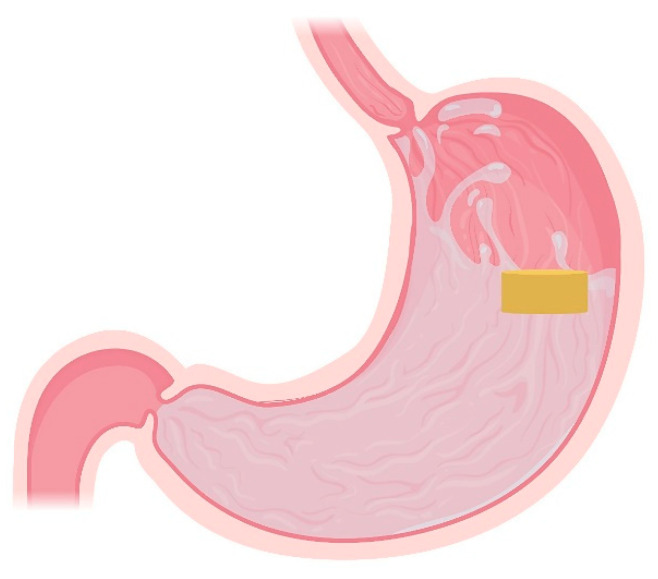
Intragastric location of the floating drug delivery systems. The figure was created using BioRender (https://www.biorender.com/) (Accessed 3 August 2021).

Floating systems are the most extensively studied gastroretentive dosage forms. The bulk density of such formulations is lower than 1.004 g/cm^3^ which enables its buoyancy in the gastric fluids for an extended period while the drug is released (Figure 2) [5,10,41,70,71]. A major advantage of this approach is that dosage form floatation results in their presence not affecting the gastric motility or causing harm to the gastric mucosa [21,72]. This category includes non-effervescent floating systems, such as hydrodynamically balanced systems and non-effervescent tablets, effervescent floating systems and raft-forming systems.

#### 4.1.1. Non-Effervescent Floating Systems

In non-effervescent floating systems, highly swellable or gel-forming polymers are employed to achieve floatation. This category includes hydrodynamically balanced systems, floating tablets (single-layer or bilayer), and low-density systems (e.g., microballoons) [10]. The use of non-effervescent floating systems comes with notable advantages, including independence of the floating mechanism of variable gastric pH and avoiding problems in patients with achlorhydria. Furthermore, the exclusion of acidic and/or basic gas-generating agents can ensure a better stability of acid- or base-labile drugs, respectively [13].

- Hydrodynamically Balanced Systems

Hydrodynamically balanced systems (HBS) are single-unit dosage forms composed of one or more gel-forming hydrophilic polymers. The key element in the development of this form is the appropriate selection of hydrophilic polymer(s) to ensure adequate flotation and release of the drug [45]. These include HPMC, hydroxypropylcellulose (HPC), sodium carboxymethylcellulose, hydroxyethylcellulose, chitosan, tamarind, xanthan, carrageenan, agar, and alginic acid. These systems are mainly formulated in gelatin capsules [73,74,75,76]. In this approach, the drug is mixed with the gel-forming polymer which hydrates and swells upon contact with the fluids, thus maintaining the density of the system below 1 g/cm^3^ [10]. Hydrophilic polymers such as HPMC, PEO, HPC and cellulose acetate phthalate are the most commonly used. Madopar^®^ HBS is a typical example of an HBS capsule, it contains a combination of levodopa and benserazide and is used for the treatment of Parkinson’s syndrome. HPMC is used as the gel-forming polymer and hydrogenated vegetable oil as a low-density fatty excipient. Valrelease^®^ is another example that contains diazepam as the API in a similar formulation and is used in patients suffering from anxiety, muscle spasms and seizures.

Since HBSs are single-unit dosage forms and gel-forming hydrophilic polymers are major components of this type of formulation, HBSs are by default controlled-release systems, as well as being gastroretentive. Hydrophilic polymers are used alone or in combinations to ensure tablet floatation and retard drug release. Dorożyński et al. developed a L-dopa HBS capsule formulation consisting of HPMC and different grades of carrageenan. It was shown that, while carrageenans were unable to retard the release of the API from the capsules for the desirable timeframe, they were able to provide flexibility in the properties of the polymeric matrices with the potential for developing tailor-made systems [45]. For controlled-release purposes, hydrophobic polymers and substances can also be included in HBS formulations to further retard the release of drugs. These include ethylcellulose or liquid paraffin [77]. Here, the increased retardation of drug release may be due to a reduced solvent penetration into the matrix due to the hydrophobic polymer, leading to reduced drug diffusion [78,79]. Furthermore, the inclusion of fatty excipients, such as hydrogenated vegetable oil, as well as giving low-density formulations also reduce the penetration of water, and thus reduce matrix erosion [80,81,82].

**Figure 3:**
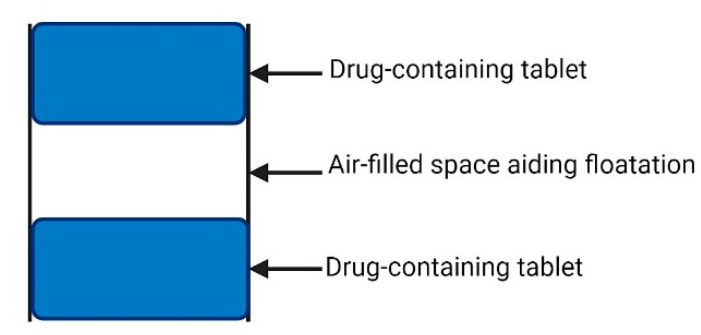
The polypropylene cylinder system developed by Krögel and Bodmeier [84]. The system consisted of entrapped air surrounded on both sides by drug-containing tablets. The air-filled space ensured a low density of the system, thus enabling its floatation. The figure was created using BioRender (www.biorender.com) (Accessed 2 August 2021).

A drawback of HBS is that it is not possible to change the release kinetics of the drug without changing the floating behaviour of the system since it is a matrix comprised of a mix of the drug and low-density hydrophilic polymers [10]. As floatation behaviour is passive and has a strong dependence on the air sealed in the dry mass centre and the swelling of the polymer surface, changes in polymer amount significantly affect system behaviour [76]. To address this limitation, strategies have been proposed to improve the efficacy of HBS. Oth et al. developed a bilayer capsule formulation of misoprostol consisting of a buoyancy layer and a drug-containing layer. Both layers contained swellable polymers and this separation allowed for their independent optimisation. *In vivo* gamma scintigraphy studies were conducted and the capsules achieved gastric retention that lasted longer than 3 h after a single breakfast meal and longer than 10 h after administration of successive meals [83]. Additionally, Krögel and Bodmeier developed a system that consisted of an impermeable polypropylene cylinder that contained entrapped air in the centre, surrounded on both ends by matrix layers containing drug and HPMC (Figure 3) [84].

- Non-Effervescent Tablets

Single-layer and bilayer floating tablets are another category of non-effervescent floating systems. For gastric retention, usually hydrophilic polymers such as HPMC, HPC, sodium alginate, PEO are used [13,17,85,86,87].

Non-effervescent single-layer floating tablets have been reported in the literature and their use is associated with novel work. Like HBS, single-layer floating tablets combine both gastroretentive and controlled-release properties since the polymers are responsible for both tablet floatation and controlled drug release. Glucophage^®^ XR is an example of this type of gastroretentive formulation that contains metformin and is used to treat type 2 diabetes. 

Hwang et al. developed highly porous cilostazol gastroretentive tablets using a manufacturing method that included a sublimation step. Menthol was incorporated into the tablets as the sublimating agent and, after compression, the tablets were placed in a vacuum oven for 12 h, so that sublimation may occur. The resultant highly porous formulations floated immediately without any lag time, while the percolation threshold of HPMC was an important factor in determining the release mechanism of cilostazol [14]. Other formulation excipients can also affect the buoyancy behaviour of non-effervescent single-layer tablets. Kim et al. developed pregabalin gastroretentive tablets containing HPMC, HPC and PEO as swelling hydrophilic polymers and demonstrated that the selection of a super-disintegrant, such as crospovidone, resulted in a prolonged tablet buoyancy, compared to using microcrystalline cellulose (MCC). The super-disintegrant is thought to have contributed to a significant expansion of the tablet volume upon contact with the dissolution media, compared to MCC which, due to its relatively weak disintegrant properties, absorbed water and hindered buoyancy [13]. 

Apart from hydrophilic polymers, hydrophobic materials and proteins, such as zein (an amphiphilic protein derived from corn), have also been used for gastric retention [88]. Raza et al. prepared single-layer tablets based on zein. L-menthol was used as a sublimating agent to provide tablets with inherent low density. The optimised formulation was able to float immediately and for 24 h in hydrochloric acid, whilst releasing the incorporated drug over the entire period. Furthermore, despite its high porosity, it was able to maintain a high mechanical strength after 12 h of dissolution [88]. Single-layer tablets containing stearyl alcohol as a floating-assistance material due to its low bulk density have also been reported [1,85], although sticking problems may hinder development [1]. In general, tabletability is a factor that needs to be considered when using fatty excipients [1]. 

Non-effervescent formulations have also been reported in the form of bilayer tablets. Bilayer tablets can be fixed-dose combinations (FDC) where a single dosing unit can contain two or more APIs in different layers [89]. Additionally, one layer can be an immediate-release layer of a drug, while the other is a sustained-release layer of the drug which is also responsible for the floating of the tablet. In other cases, the tablet can be comprised of a drug-free gastroretentive layer and a sustained-release drug layer [21]. To avoid long floating lag times, low-density materials are used in the gastroretentive layer to achieve rapid buoyancy of tablets. Additionally, rapid buoyancy can result from low compression force values that allow for higher tablet porosity and lower tablet density and hence a rapid polymer wetting and swelling that further reduces the density of the dosage form [17]. Furthermore, materials such as camphor and L-menthol that can undergo sublimation may be used. These materials are sublimed upon tablet heating, leading to an increased tablet porosity and reduced density that enables tablets to float immediately [14,88,90]. Among these materials, camphor is the most frequently used. Nguyen et al. prepared gastroretentive bilayer tablets based on Kollidon^®^ SR which was used in the gastroretentive layer along with camphor as a sublimation agent. Due to the inherently low density of Kollidon^®^ SR and the improved wet strength of the gastroretentive layer linked to the presence of a hydrophobic polymer, along with the further reduction in the tablet density from camphor sublimation, a 12-h floating was successfully reached *in vitro*, even at a rotation rate of 200 rpm [72]. This high paddle rotation speed is designed to create conditions that better simulate the *in vivo* conditions in fasted Beagle dogs, compared to typically used lower rotation speed values [72,91].

- Low-Density Systems

Most floating gastroretentive formulations tend to have a lag time prior to floating in stomach contents. During that time, a premature evacuation of the dosage form from the stomach may take place via the pyloric sphincter [9]. Low-density systems are formulations that have an inherent density lower than 1.004 g/cm^3^ which enables their immediate floatation in the stomach. These include drug-loaded microballoons/hollow microspheres, sponges and are multiple-unit floating systems [92,93].

Microballoons have a low-density, hollow core, and are made of materials that can entrap oil or air [94,95,96,97,98]. Commonly used polymers in these systems are polycarbonate, calcium alginate, Eudragit^®^ S, cellulose acetate, agar and low-methoxylated pectin. They can be prepared through simple solvent evaporation or solvent diffusion techniques [75]. Kawashima et al. prepared microballoons based on polyvinyl alcohol (PVA) and Eudragit^®^ S using an emulsion-solvent diffusion technique [94]. Eudragit^®^ S formed a mechanically strong film (shell) on the outer surface of the microballoons which ensured the stability of the system during solvent evaporation and hindered drug release at pH values below 7, due to solubility and permeability limitations of the polymer. The drug (tranilast or ibuprofen) was loaded into the outer shell of the microballoon. Around 90% of the microballoons floated on the surface of the hydrochloric acid medium, however, microballoons that lost buoyancy could pass from the stomach and still release the drug in a controlled manner in the upper small intestine which was the absorption site. Therefore, this multiple-unit system seemed to be versatile in achieving a prolonged residence time in the stomach, whilst providing a controlled drug release and ensuring a good bioavailability [94]. Furthermore, Ammar et al. prepared cinnarizine microballoons based on cellulose acetate butyrate (CAB), a hydrophobic polymer that had not been used for this purpose before [99]. Due to the hydrophobic nature of the drug, it was able to be incorporated into the formulation in a sufficient amount. The *in vitro* release testing demonstrated a biphasic release of cinnarizine from the formulation that was probably due to availability of the drug on the surface of microballoons as well as the interior of the matrix. After 1 h, the drug release rate slowed and was followed by a controlled-release phase linked to slow drug diffusion through the microballoon polymer structure. Increasing CAB concentration levels led to slower drug release rates, probably due to a more compact polymer structure, as well as poorer wetting of the microballoons derived from the hydrophobic nature of the polymer. Finally, a clinical trial in humans demonstrated a significantly prolonged release of cinnarizine *in vivo*, alongside higher AUC_0−24h_ and AUC_0−∞_ values and plasma concentration levels over 24 h, compared to commercial Stuval^®^ tablets. These data illustrate the gastroretentive capability of the microballoon formulation, as well as the importance of the microballoon small particle size in achieving high drug bioavailability, compared to the monolithic tablet formulation [99].

**Figure 4:**
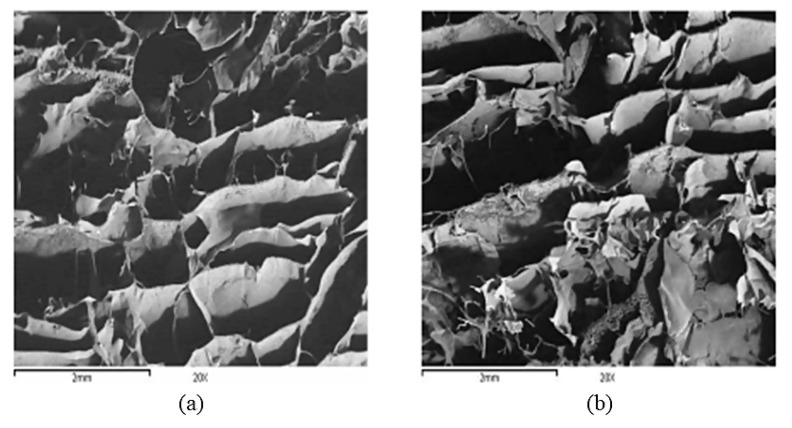
Scanning electron microscopy (SEM) images demonstrating the porous structure of the gastroretentive sponges before (**a**) and after (**b**) compression. The compression did not seem to damage the porous structural framework of the sponges which most likely accounted for their zero floating lag time. Reprinted from International Journal of Pharmaceutics Vol 472, Tadros and Fahmy, Controlled-release triple anti-inflammatory therapy based on novel gastroretentive sponges: Characterization and magnetic resonance imaging in healthy volunteers, Pages 27–39, Copyright 2014, with permission from Elsevier.

Tadros et al. developed lornoxicam gastroretentive composite sponges based on an interpolymer complexation (IPC) between chitosan and chondroitin sulphate (Figure 4). The drug was dissolved and dispersed in the polymer solution and lyophilisation was applied to prepare the sponges. The sponge structure was highly porous and ensured their immediate floatation in hydrochloric acid with zero lag time. Furthermore, the IPC sponge system proved to be suitable in controlling the release of lornoxicam in hydrochloric acid for more than 12 h [100]. Interestingly, biphasic release profiles were recorded which were interpreted as per the “sequential layer” model [101]. This concluded that the biphasic release was due to water imbibition into the matrix and drug diffusion through the formed gel layers for the first 4 h, followed by increasing polymer erosion and drug release for up to 12 h of dissolution. The drug release was dependent on the chitosan:chondroitin sulphate ratio which indicated that the formulation could be altered to achieve tailored release profiles to meet individual patient needs [100]. 

There are certain limitations to the application of low-density gastroretentive systems. Since they are multiple-unit systems, the release rate can be different between different doses and units. Furthermore, if system integrity is compromised, a consequent burst release of drug could lead to adverse effects since drug loadings tend to be higher in controlled-release formulations [92].

#### 4.1.2. Effervescent Floating Systems

Effervescent systems contain a gas-forming agent and/or volatile liquids that contribute to their floatation. In a gas-generating floating system, swellable polymers are mixed with effervescent agents such as sodium bicarbonate, calcium carbonate, tartaric acid, and citric acid used alone or in combinations [10,16,102]. Upon contact of the system with gastric fluids, the gas-generating agent reacts with hydrochloric acid and CO_2_ gas is generated. CO_2_ causes the floatation of the tablet and helps maintain tablet buoyancy by getting trapped in the hydrocolloid matrix of the system. It also influences drug release properties [1,53,103,104]. One disadvantage of effervescent systems is the fact that, unless low-density materials are used, they show long floating lag times if the generation of gas bubbles that will promote buoyancy is not fast enough and are, therefore, under the risk of premature gastric emptying [105]. Furthermore, they are not suitable to be administered in patients with achlorhydria since the low excretion of gastric acid in these patients can lead to a higher gastric pH, thus resulting in extended floating lag times of the effervescent systems which can be problematic for their gastric retention [13].

Effervescent floating systems can be single-layer and bilayer floating tablets and multiple-unit systems [10,17,41]. Single-layer tablets can be prepared by mixing the drug with the gas-generating agent(s), polymer(s) and other excipients. In bilayer tablets, different strategies can be followed. One layer may contain the drug, polymer(s), and gas-generating agent(s), whereas the other layer could constitute an immediate-release one consisting of drug and excipients without gas-forming agents and release-retarding polymer(s) [10]. The amount of a gas-generating agent, such as sodium bicarbonate, can significantly affect the total hydration volume of a tablet, along with its floatability. The higher the percentage of gas-generating agent, the greater and faster-growing hydration volumes can be, along with reduced floating lag times [17]. Additionally, the presence of sodium bicarbonate can overcome tablet floatability issues in cases where high tablet compaction forces result in low tablet porosity values that are insufficient for floatation [17]. Additionally, in other cases, one layer consists of the drug, release retardant(s) and other excipients, while the other layer is the gastroretentive layer containing the gas-generating agent(s) and hydrophilic polymer(s) [86]. Cifran^®^ OD is a ciprofloxacin commercial product that constitutes effervescent single-layer gastroretentive tablets coated with a polymer film that is responsible for tablet buoyancy.

Diós et al. assessed the effect of the relative amounts of sodium alginate, L-HPC B1 and sodium bicarbonate on different buoyancy parameters, including floating lag time, maximal floating force, maximal floating force calculated to 100 mg tablet weight mass, time needed for maximal floating force and drug release [106]. The effect on each parameter was calculated using mathematical models and the optimised floating drug delivery compositions were successfully prepared and assessed *in vivo*. The optimised tablets demonstrated superior floating behaviour and biphasic release, compared to two commercially available metronidazole products. The formulation was retained for 8 h in rat stomach, as determined by X-ray CT [106].

Apart from hydrophilic polymers, in other cases, a hydrophobic protein, such as zein, has been used in effervescent tablets for gastric retention. Raza et al. reported the preparation of zein-based compression coated single-layer tablets. The tablet core contained zein matrix as a release retardant, while the compression coating was comprised of zein and sodium bicarbonate. The tablets showed a short floating lag time and a total floating time longer than 12 h, along with a 12-h controlled release of captopril [104]. Increasing amounts of sodium bicarbonate accelerated drug release rate from the matrix, probably due to higher pore formation that allowed for increased penetration of the dissolution medium into the matrix. The floating mechanism was a combination of rapid formation of pores the size of which grew over time (confirmed via scanning electron microscopy and porosity studies) and CO_2_ entrapment in the swelling matrix [104].

Recently, studies have reported the use of nanofibre technology for gastric retention purposes. Nanofibres are generally produced via electrospinning and can be tuned to provide controlled release of drugs through different mechanisms [107,108,109,110]. Nanofibres can offer specific benefits as part of an effervescent GRDDS. These include their high porosity that can promote the penetration of gastric hydrochloric acid into the effervescent film within the nanofibre, as well as their ability to entrap the generated CO_2_ gas which, not being able to diffuse out of the system, significantly prolongs the floatation of GRDDS [111]. Furthermore, alongside their high porosity, the high surface area-to-volume ratio of nanofibres results in an intrinsically low bulk density which helps ensure their immediate buoyancy upon contact with the gastric fluids [112]. Tort et al. reported the development of self-inflating effervescent nanofibre membranes containing pramipexole as the API [113]. A mixture of Eudragit^®^ RL and RS polymers was employed to control drug release kinetics, while the effervescent films consisted of PEO and sodium bicarbonate and were embedded into the nanofibre membranes. Due to their low bulk density, the nanofibres floated immediately in simulated gastric fluid (SGF). Furthermore, the study demonstrated that the presence of CO_2_ gas generated in the membrane structure seemed to be crucial for prolonged floatation of the nanofibres since the effervescent nanofibres floated longer than 72 h, while the non-effervescent nanofibre membranes did not float effectively. In terms of *in vitro* drug release, the presence of Eudragit^®^ RS ensured minimum burst release from the nanofibres. The release of pramipexole was controlled over 24 h and mainly occurred via drug diffusion, as demonstrated by fitting of the release data to the Korsmeyer-Peppas model [113]. 

Multiple-unit effervescent floating systems may consist of sustained-release cores surrounded by one or more layers. The layer(s) can contain gas-forming agents along with polymers with swelling properties [10,41,53].

#### 4.1.3. Raft-Forming Systems

Raft-forming system is comprised of effervescent excipient(s) and gel-forming polymers to achieve floatation and a sustained release of drugs. These systems are tablets or liquids at room temperature that can undergo gelation when in contact with gastric fluids, due to an increased temperature, or as a response to pH change. Therefore, their behaviour can be either temperature-dependent or characterised by cation-induced gelation. Either way, the formation of a gel that is thick enough to remain intact for hours within the stomach contents leads to their buoyancy and controlled drug release applications [41,114,115]. This system is mostly used to achieve localised effects. Floating rafts can act as a blockade between the oesophagus and the stomach. This property renders these formulations ideal for the management of gastric oesophageal reflux disease. Furthermore, when an antacid is incorporated into the raft-forming system in significant amounts, the system can aid in the treatment of both indigestion and heartburn via acid neutralisation in both the stomach and the oesophagus [116,117]. Upon contact with the gastric fluids, these systems swell and form a viscous cohesive gel layer termed a raft [10,41,118]. Raft systems are used for the delivery of antacids, such as simethicone, aluminium hydroxide and calcium carbonate. However, the mechanical strength of the raft is weak, which could result in its premature disruption by the MMC [114,118]. Gaviscon^®^ is a commercial raft-forming formulation that is available as tablets or liquids. It is used to treat indigestion and heartburn. Its raft-forming mechanism is based on a combination of sodium alginate, sodium bicarbonate and calcium carbonate. Topalkan^®^ is an antacid formulation containing aluminium and magnesium as active components, while alginic acid is the raft-forming excipient.

Fabregas et al. prepared an antacid raft-forming system that contained almagate as the active component. Sodium alginate was used as the raft-forming polymer and sodium bicarbonate and acid neutraliser as effervescent agents [119]. The presence of the effervescent agents contributed to the prolonged gastric residence of the novel system which therefore proved to be promising as an antacid formulation [119]. Nabarawi et al. prepared a controlled-release floating raft system that contained mebeverine as the API. A combination of hydrophilic and lipid polymers was used in the formulation and the system was characterised for its floating behaviour and *in vitro* drug release [120]. A viscous and cohesive gel was formed upon polymer swelling that entrapped CO_2_ bubbles generated by the reaction of carbonates and hydrochloric acid [9]. Drug release occurred via a Fickian diffusion mechanism. A pharmacokinetic study of the optimised raft system in Beagle dogs demonstrated that the formed raft remained intact for more than 12 h in the Beagle dog stomach promoting the sustained release of the antacid [120].

Wannasarit et al. reported the development of a gastroretentive raft-forming system based on Eudragit^®^ EPO-*Centella asiatica* extract-solid dispersions containing the poorly soluble asiaticoside and madecassoside which have proven to be effective against gastric ulcers [121,122]. The solid dispersion approach proved to be able to enhance the aqueous solubility of the glycosides, while the presence of HPMC K100M in the raft ensured a sustained release of the compounds over 8 h with the release kinetics and mechanism being controlled by Fickian diffusion for both glycosides. Furthermore, the raft strength was higher than 7 g and hence could be considered strong and coherent and be able to withstand the peristaltic movements of the gastrointestinal tract [122,123]. 

While liquid is the most common formulation form for raft-forming systems, recently, Hanif et al. reported the development of gastroretentive raft-forming tablets containing ibandronate. Nanosized citrus pectin (NCP) was prepared and used as the raft-forming polymer in the tablets, due to its gelling, antacid and antiulcer properties [124]. When combined with calcium carbonate, NCP demonstrated the highest gel strength, due to a cross-linking pattern of the raft through the calcium ions [125]. The tablets demonstrated a rapid release of ibandronate that was completed within 20 min, potentially due to rapid diffusion of the drug through the foam structure of the raft that was ought to the incorporation of NCP into the formulation. Furthermore, an *in vivo* study in albino rats demonstrated an enhanced bioavailability of ibandronate in the rats, compared to a reference formulation, potentially due to the effective penetration enhancement of PEG 400 [124].

In general, the use of floating systems comes with certain potential issues. These include the possibility that system units may stick together (for multiple-unit systems) or be obstructed in the GIT, which could cause gastric irritation. Therefore, drugs that may have an irritating effect on the gastric mucosa are not considered suitable candidates for floating systems [10,41,59].

### 4.2. High-Density Systems

**Figure 5:**
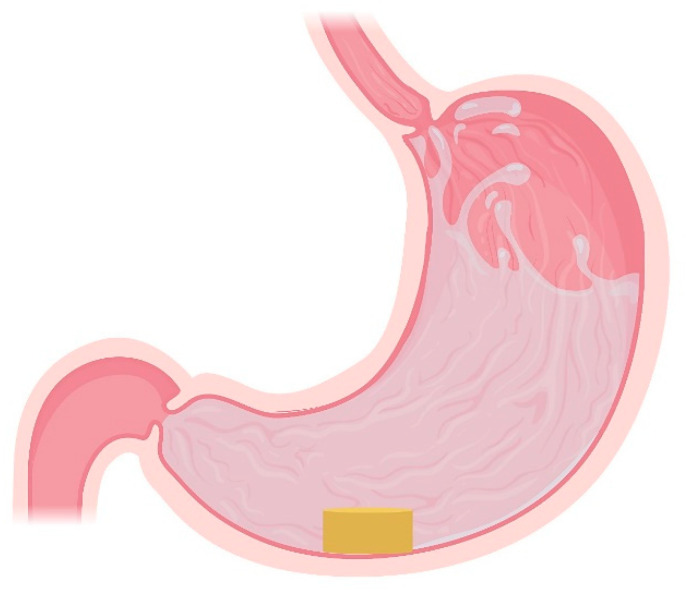
Schematic localisation of high-density systems in the stomach. The figure was created using BioRender (www.biorender.com) (Accessed 3 August 2021).

High-density systems have a density greater than that of gastric fluids (1.004 g/cm^3^) and therefore sink to the bottom of the stomach fluids and achieve gastric retention, probably via increased resistance to the gastric contractions that is linked to their high density (Figure 5) [54]. Excipients that are commonly used to ensure a sufficiently high density of these systems include barium sulphate, zinc oxide, iron oxide, iron powder, and titanium dioxide. Usually, the dosage forms reported in the literature are high-density pellets or tablets [126,127].

Sharma et al. reported the preparation and characterisation of gastroretentive high-density pellets containing zero-valent iron nanoparticles (ZVINPs) [127]. Barium sulphate was used as the high-density component, while Carbopol^®^ was incorporated as the release retarding agent. The sinking time test demonstrated an immediate sinking of the optimised pellets, while they were able to retard the release of iron over 19 h *in vitro*, due to the presence of Carbopol^®^. An *in vivo* study was conducted in male Wistar rats and the X-ray analysis showed that the pellets were retained in the stomach over 10 h, while the plasma concentration of iron remained at high levels over 24 h with minimum fluctuations that can be attributed to the controlled release of iron from the pellets [127].

Desai et al. developed a novel multiparticulate pulsatile high-density tablet containing clopidogrel. The aim was to prepare tablets that would be retained in the stomach for 7–8 h and release the drug after a predetermined lag time [126]. Iron oxide was used as the density-increasing agent and high-density pellets were prepared using extrusion and spheronisation, followed by their compression into tablets. HPMC K4M and ethylcellulose were used as coating materials to achieve a lag time of 6 h, followed by a burst release of clopidogrel. The *in vitro* release results demonstrated a rapid release of the API after a 6-h lag time. Furthermore, an *in vivo* X-ray study in albino rabbits demonstrated 8-h retention of the tablets in the animal stomach. Therefore, this combination of multiparticulate pulsatile and high-density gastroretentive systems demonstrated a successful contribution towards a chronotherapeutic effect of clopidogrel [126].

Even though such systems are promising in achieving long gastric residence times (GRTs), it is difficult to design high-density pellets containing high doses of drugs. Furthermore, the clinical significance of these systems is yet to be proven since only few animal studies have been carried out [10,63].

### 4.3. Mucoadhesive/Bioadhesive Systems

**Figure 6:**
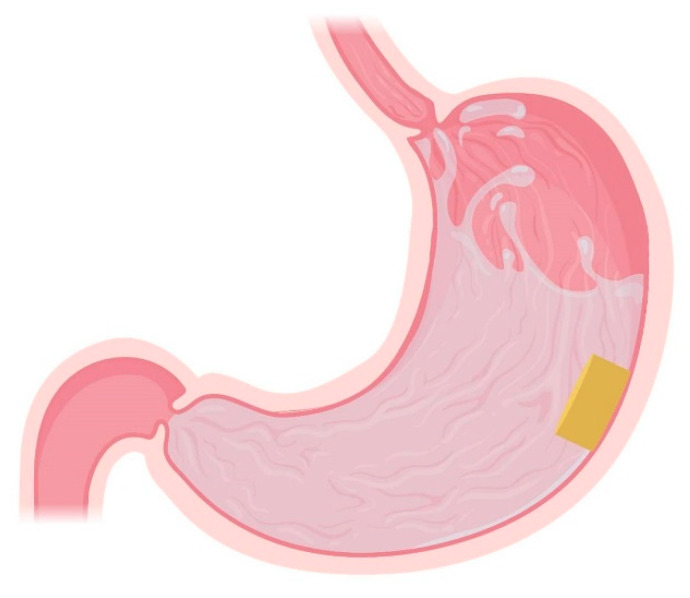
Localisation of mucoadhesive/bioadhesive drug delivery systems in the stomach. The figure was created using BioRender (www.biorender.com) (Accessed 3 August 2021).

Adhesion is the process by which two surfaces are “fixed” to one another [128]. When adhesion happens in a biological setting, it is referred to as “bioadhesion”, while if it occurs on mucosal surfaces it is termed “mucoadhesion” [30]. Regarding gastric retention applications, this system can adhere to the surface of the gastric mucosa and prolong the GRT of drug compounds (Figure 6) [20,41]. In these systems, the drug is incorporated into one mucoadhesive agent or a combination of them that can be natural or synthetic hydrophilic polymer(s). Xifaxan^®^ is a commercial bioadhesive tablet formulation containing rifaximin as an antibiotic and is used to treat traveller’s diarrhoea, hepatic encephalopathy, and irritable bowel syndrome with diarrhoea (IBS-D).

Mucoadhesion is achieved through bond formation between the swelling polymer and mucous surface [31]. This is a process consisting of two steps, the contact stage and the consolidation stage [129]. Different theories supplementary to one another have been reported about the mechanism of mucoadhesion. These include the wettability theory, the electronic theory, the fracture theory, the adsorption theory and the diffusion-interlocking theory [30,130,131,132].

The wettability theory proposes that the adhesive component of a drug delivery system penetrates surface irregularities, hardens and subsequently anchors itself to the surface [30]. This theory applies to liquid or low-viscosity mucoadhesive systems and the critical attribute that is used to measure their adhesion performance is the wettability and spreadability of the system across the biological substrate. These parameters are dependent on the contact angle of the two surfaces. Lower water:polymer contact angles will facilitate the hydration of these systems, thus promoting the interaction between the polymer and the mucosal surface chains [133]. Additionally, mucoadhesive systems with similar structure and functional groups to the mucus layer may show increased miscibility which, in turn, leads to a greater degree of polymer spreadability across the mucosal surface [30].

The electronic theory postulates that adhesion can take place via electron transfer between the mucoadhesive system and the mucus due to differences in their electronic structures. This electron transfer generates a double layer of electrical charges at the mucoadhesive system:mucus interface that leads to the formation of attractive forces [130]. However, this theory is considered controversial since electrostatic forces are not just considered a result of high joint strength, but rather a predominant cause of adhesion [30,128].

According to the fracture theory, the adhesion strength between two surfaces is related to the force that needs to be applied to separate the two surfaces. In terms of mucoadhesion, this theory relates the force required to detach the polymer system from the mucus to the strength of the adhesive bond. The fracture work seems to be dependent on the polymer chain length and the degree of cross-linking in the polymer network. More specifically, an increase in the former or reduction in the latter increases the work of fracture [134].

The adsorption theory suggests that adhesion could be the result of different surface interactions between the adhesive polymer and the mucus, termed primary and secondary bonding. Primary bonding includes ionic, covalent and metallic bonds which are generally strong and persistent and are, therefore, not desirable [28,30,128,135]. Secondary bonding includes mainly van der Waals interactions, hydrophobic and hydrogen bonds [28,135]. Even though these interactions require less energy to break, they are considered the most prevalent in terms of surface interactions in mucoadhesion processes, probably because they are semi-permanent bonds [128,131,134].

The diffusion-interlocking theory refers to a time-dependent diffusion of the adhesive polymer chains into the glycoprotein network of the mucus. This is a two-way process since the polymer networks diffuse into one another with the penetration rate depending on the diffusion coefficients of both inter-penetrating polymers. The factors that most significantly affect this polymer interaction are molecular weight, cross-linking density, chain mobility and expansion capacity of each of the polymer networks [132,136,137,138]. Additionally, temperature is an environmental factor that can affect this process via an effect on the friction coefficients of the polymers [139]. It is suggested that, for a successful interpenetration and chain entanglement at a molecular level to occur, a minimum chain length of 100,000 Da is required [30,140,141]. The effectiveness of adhesive polymer chains to interact, diffuse, interpenetrate and achieve inter-locking with the polymer chains of the surface mucus increases with their length. Furthermore, increased cross-linking density results in reduced polymer chain mobility, thus leading to a decline in interpenetration [140]. In addition to the above, the miscibility of the two polymer systems with one another is very important in achieving effective interpenetration. Therefore, maximum diffusion and adhesion can be achieved when the solubility values of the adhesive and mucus polymer networks are similar [135].

Many different mucoadhesive dosage forms have been reported in the literature, including beads, microspheres, films, capsules and tablets [28]. Polymers such as chitosan, sodium alginate, HPMC, polyethylene glycol and polyacrylic acid have mucoadhesive properties that render them suitable for use in these systems [9,41]. These polymers bind to the mucosal surfaces and, thus, maintain the drug in continuous contact with the mucus and increase the residence time of the drug at the application site. These polymers should be inert, non-irritating to the mucosa, non-toxic, adhering to the mucosal surface and site-specific [10].

Chitosan hydrogels have been used extensively in gastroretentive drug delivery system applications. Chitosan can form gels with improved mechanical strength through chemical cross-linking using glutaraldehyde, glyoxal or other substances as cross-linkers, or through physical cross-linking as ionically cross-linked chitosan gels with multi-valent phosphates or as polyionic complexes of positively charged chitosan with a negatively charged polymer, such as alginate or polylactic acid. Chitosan-based hydrogels have been used for mucoadhesive and other gastroretentive, as well as controlled-release, applications [47,142,143,144,145,146,147,148,149,150,151,152,153]. 

Poly(acrylic acid) (PAAc) and its derivatives have been reported as excellent mucoadhesive agents [48]. Sarkar et al. developed metformin mucoadhesive tablets where a graft-copolymer of PAAc with gellan gum (GG) was used as the adhesive agent. GG was used as the main polymeric backbone and PAAc was grafted onto it [19]. After the synthesis was completed, PAAc-g-GG-based tablets were prepared, alongside GG-, HPMC K15M- and Carbopol^®^-based ones. The viscosity of polymer solutions formed by the PAAc-g-GG graft-copolymers with the maximum amount of grafting was found to be the highest which was expected since branched polymers tend to have higher viscosity values than linear chain polymers. In terms of *in vitro* drug release, the tablets containing copolymers with a high degree of grafting demonstrated a sustained release of metformin over 10 h, likely due to their comparatively denser network leading to slower water uptake and network relaxation. The drug release from most of the formulations followed Higuchi and Korsmeyer-Peppas kinetics, alongside a Fickian-diffusion-based mechanism of drug release. Finally, regarding mucoadhesion, GG-only tablets showed no such capability, while the PAAc-g-GG tablets demonstrated a strong mucoadhesion on goat stomach mucosa *ex vivo*, based on their long mucoadhesion time and high adhesion strength. This is probably due to numerous –COOH groups that can form multiple hydrogen bonds with the mucus molecules under an acidic environment that favours interaction of these groups with the mucin rather than the surrounding water. Therefore, PAAc-g-GG-based tablets are promising in improving the oral bioavailability of drugs with a narrow absorption window, such as metformin [19].

Nanoparticles are in general known to suffer from a rapid passage through the stomach and intestine, and thus being unable to release drugs in a controlled manner and to an acceptable extent [20]. This limits their oral applications. To address this challenge, Sarparanta et al. developed thermally hydrocarbonised porous silicon (THCPSi) nanoparticles [20]. Porous silicon was used since it is known for its biocompatibility and biodegradability, as well as variable particle size, pore size and degree of porosity [154,155]. The nanoparticles were coated with hydrophobin class II which ensured the efficient dispersal of the nanoparticles in aqueous media with minimal aggregation. During an *in vitro* study on human gastric adenocarcinoma (AGS) cells, the nanoparticles demonstrated a gradual adhesion to the cells over time with 35% of the nanoparticles being retained for at least 4 h on the cells. However, it took 30–60 min for the nanoparticles to be attached to the cells with the mucoadhesion achieved probably via hydrophobic and electrostatic interactions. Nevertheless, an *in vivo* biodistribution study in rats demonstrated that the nanoparticles were able to be retained in the rat stomach for 3 h, prior to their emptying from the stomach towards the duodenum, where the hydrophobin class II coating would be expected to be shed, due to the presence of surfactants [20]. 

The use of mucoadhesive systems comes with certain limitations. Site targeting can be challenging, due to differences in the composition of the mucus across the mucosal membrane. Furthermore, the constant turnover of the mucus, along with high stomach hydration, can contribute to a reduced bioadhesion of the systems. Finally, there is a risk of adhesion of the system to the oesophagus which could result in collateral lesions [9,56].

### 4.4. Expandable Systems

Expandable drug delivery systems are designed to grow in size or reconfigure their geometry within the stomach to prevent passage through the pyloric sphincter and have a longer GRT [15]. These systems need to have a small size for easy oral intake, expand in the stomach and attain dimensions larger than the diameter of the pyloric sphincter and have their size reduced after drug release is completed, for them to be evacuated from the stomach [5,9,15]. Due to their ability to block the pyloric sphincter, these systems are also referred to as “plug-type” systems. It has been suggested that these systems should expand to a size greater than 15–16 mm in the fasted state and greater than 12–13 mm in the fed state to ensure successful gastric retention, based on the resting orifice of the pyloric sphincter (12.8 ± 7 mm) [156,157]. Furthermore, a sufficiently high tablet wet strength is an additional requirement that will ensure the protection of gastroretentive tablets from premature rupture and emptying under the effect of the housekeeper waves [158]. The expansion takes place through swelling and/or unfolding which allows for volume and shape modification, respectively [15,159]. Accordion Pill^®^ Levodopa/Carbidopa is a commercial expandable capsule formulation containing carbidopa and levodopa as active components and is used to treat Parkinson’s disease. It achieves gastric retention via an unfolding mechanism through the presence of folded multilayer polymeric films. Additionally, Acuform^®^ and Geomatrix^®^ are two expandable system technologies that have been used in commercial gastroretentive formulations, such as Requip^®^ XL (ropinirole), Glumetza^®^ (metformin), Nucynta^®^ ER (tapentadol), Gralise^®^ (gabapentin) and Janumet^®^ XR (Sitagliptin and metformin).

Hydrophilic polymers, such as HPMC, PEO and Carbopol^®^, are usually utilised in the swelling expandable systems due to their ability to absorb water and increase the system volume by swelling. Likewise, in unfolding systems, the drug and polymer can be in a folded/compressed state inside a gelatin capsule. When the capsule comes in to contact with the gastric fluids, it dissolves, and the mechanically preferred expanded configuration is released. In these systems, the critical attributes that need to be considered include the molecular weight, viscosity, swelling or expanding properties and biodegradability of the polymer chosen to maintain gastric retention and sustained release of the drug [9,160].

**Figure 7:**
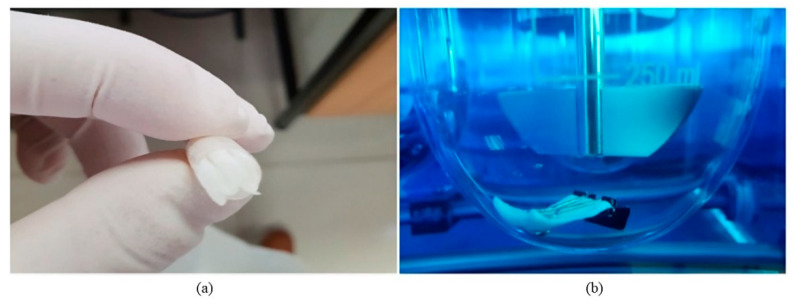
Transition of the gabapentin-loaded polymer layer from the folded (**a**) to the expanded (**b**) state during *in vitro* release testing [161]. The present figure combines Figures 11 and 12 from the original research paper [161].

Rimawi et al. developed an expandable matrix system in the form of a layer that contained gabapentin as the API. Gelatin was used as a swellable hydrophilic polymer which increased in size upon contact with the body fluids, as well as a plasticizer (Poloxamer 407) that increased the flexibility of the layer, and hydrophobic polymers (Eudragit^®^ L100, L100–55 and S100) as release retarding agents [161]. The system was able to unfold and expand within 15 min to a diameter larger than 20 mm after contact with hydrochloric acid dissolution medium which could potentially prevent a premature evacuation of the system from the stomach (Figure 7). The unfolding mechanism involved a specific combination of citric acid and sodium bicarbonate trapped in-between the folded layer parts. When activated, it generated carbon dioxide gas that helped push the folded parts away from each other. Furthermore, the release of gabapentin was retarded for longer than 6 h and followed zero-order kinetics [161].

Xanthan gum has shown promising properties as a swelling and expanding agent either used solely or in combination with other materials, such as guar gum [162]. In a study, xanthan gum was compared against sodium alginate, gellan gum and pectin in the development of levofloxacin gastroretentive tablets, regarding the polymer ability to swell and retard drug release. Xanthan gum was able to swell rapidly and maintain a thick gel over 24 h, whilst releasing levofloxacin over 8 h. The release of levofloxacin from the optimised tablets followed Weibull kinetics with a non-Fickian diffusion mechanism [156].

Bellinger et al. developed an ultra-long-acting ivermectin gastroretentive expandable system. The system was a stellate dosage form consisting of six arms joined at a central core made of an elastomeric material. The arms consisted of solid dispersions of the drug based on a linear polycaprolactone (PCL) polymer and Pluronic^®^ P407. The base of the system that was responsible for the unfolding mechanism consisted of a PCL-based polyurethane (PU) thermoset elastomer. This material can undergo a high degree of strain without tearing, expand rapidly within 5 to 30 min after removal from the capsule and remain deformed within the capsule without risk of plastic deformation [163]. PCL is a hydrophobic polymer that swells minimally in water and is stable in strongly acidic conditions. The dosage form was able to control the release of ivermectin over longer than 2 weeks both *in vitro* and *in vivo* after oral administration in pigs. Drug release occurred through polymeric matrix erosion. Furthermore, enteric linkers present in different parts of the system ensured its dissociation into small pieces to ensure an eventual safe passage through the small intestine. However, due to the characteristics of the polymer matrices that are necessary to maintain a long gastric residence of the system, its application is limited only to small doses of drugs [163]. Although the efficacy of the system needs to be confirmed in humans, its application as an ultra-long-acting gastroretentive form is promising and advanced for oral drug delivery applications for the treatment of a broad range of clinical conditions [164].

**Figure 8:**
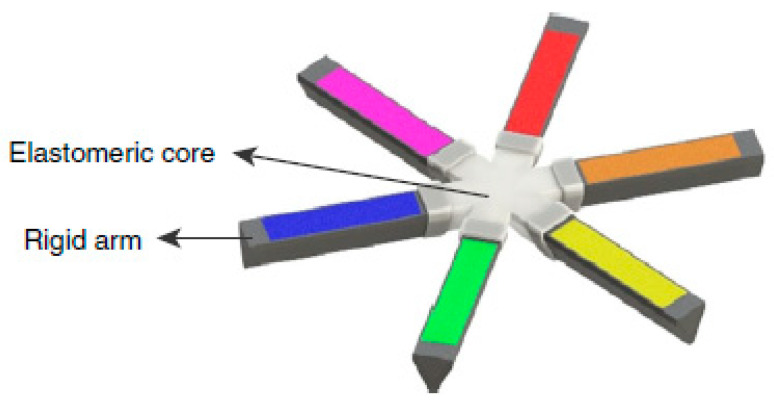
Design of the “Long-acting Pill” technology in its expanded form. The dosage form consists of a water-insoluble elastomeric core and six drug-loaded polymeric arms. Reprinted from Nature Communications Vol 9, Kirtane et al., Development of an oral once-weekly drug delivery system for HIV antiretroviral therapy, Pages 1–13, Copyright 2017, published under a Creative Commons Attribution 4.0 International License (http://creativecommons.org/licenses/by/4.0/, accessed on 2 August 2021) (the “License”). No changes were made to the original figure presented as Figure 1a in the original research paper.

Kirtane et al. employed the same concept (Figure 8) as Bellinger et al. for a once-weekly oral administration of a combination of the antiretroviral drugs—dolutegravir, cabotegravir and rilpivirine [165]. Here, the researchers created a formulation consisting of a combination of thermoplastic urethanes Elastollan^®^1185 and Elastollan^®^R6000 as the solid dispersion matrix for the drugs. This combination provided improved mechanical properties, regarding their value for maximum stress and resistance towards repetitive stomach bending forces [165]. The release of all the drugs was sustained over 7 days both *in vitro* and *in vivo* after oral administration in pigs, with their plasma concentration remaining approximately within steady-state levels over the 7-day period. By employing mathematical modelling methods to evaluate the impact of the delivery system on patient outcomes and epidemiological trends, the authors estimated a significant potential for the improvement of patient adherence to HIV treatments, thus improving patient health, whilst significantly reducing the number of new HIV infections [165]. However, the same limitations apply to this system, regarding the low doses of drugs that can be incorporated into these systems, as well as the requirements for stability of drugs in acidic pH, elevated temperatures and high humidity [163,165]. Based on the research results, the company that contributed to the development of this technology, Lyndra^®^ Therapeutics, announced a Notice of Allowance obtained from the U.S. Patent and Trademark Office (USPTO) for this technology under the name “Long-acting Pill” (US20170266112A1 patent). A once-a-week risperidone treatment for schizophrenia showed promising data in phase II clinical trials [166], while other drugs treating different clinical conditions have been formulated into the Long-acting Pill and are bound for and/or undergoing phase I clinical trials [167,168].

The applications of expandable systems come with a few limitations. There are difficulties in storing easily hydrolysable and biodegradable polymers, while manufacturing can be challenging, alongside a potential lack of cost-effectiveness. Furthermore, there can be challenges in maintaining the structural integrity of the systems in the stomach. Finally, the application of this type of system may cause adverse effects such as bowel obstruction, intestinal adhesion, and gastropathy [2,9,15].

### 4.5. Superporous Hydrogel Systems

**Figure 9:**
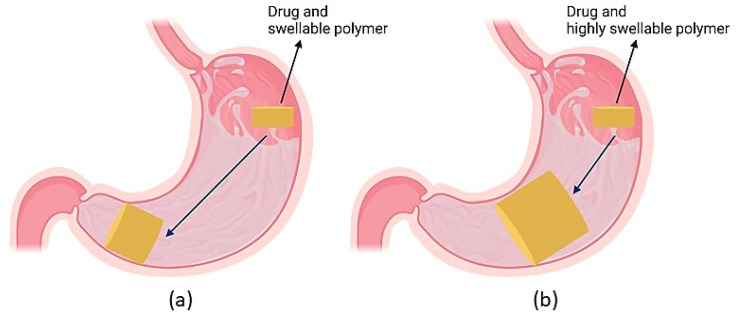
Intragastric behaviour of (**a**) expandable and (**b**) superporous hydrogel systems. The figure was created using BioRender (www.biorender.com) (Accessed 3 August 2021).

These systems have gained high popularity as controlled-release formulations, due to their high mechanical strength and elastic properties [33]. Superporous hydrogels are highly cross-linked polymers that can absorb significant amounts of aqueous fluids and swell within relatively short periods to form a stable gel [169,170]. Such systems have a pore size greater than 100μm. This enables them to rapidly absorb water by capillary wetting and swell to an equilibrium size in a short time (Figure 9) [5,169]. While the conventional hydrogel system swelling is a slow process and can result in premature evacuation from the stomach, superporous hydrogels can swell up to 100 times or more and can gain enough mechanical strength to resist pressure from gastric contractions, thereby increasing the GRT. In these systems, highly swellable polymers, such as sodium croscarmellose and sodium alginate are used [9,10,32]. However, such polymers have swelling behaviour that depends sensitively on pH changes, and unintended reversal or slowing of polymer swelling can result in poor mechanical strength of the system structure and its premature evacuation from the stomach [9,32].

Three different generations of superporous hydrogels have been reported. The first-generation superporous hydrogel systems consisted of one cross-linked polymer [169,171]. Their mechanical strength can be enhanced by the addition of other cross-linked hydrophilic polymers into the primary polymeric network which leads to the formation of so-called second-generation superporous hydrogels (superporous hydrogel composite, SPHC). This can enhance the cross-linking density of the hydrogels without rendering them too brittle [169,172]. Finally, Omidian et al. reported the development of third-generation hydrogels which were formed through the creation of a novel interpenetrating polymer network (IPN), where sodium alginate was added in the solution of a monomer (acrylamide), a cross-linker (bisacrylamide) and the other ingredients, followed by polymerisation. The polymerised superporous hydrogel was further treated with calcium chloride to promote the metal complexation of the alginate portion of the IPN. The IPN of polyacrylamide and sodium alginate was able to swell rapidly and form a gel that was mechanically strong, highly elastic and resistant to various types of forces [172]. 

Bhalla et al. also reported the preparation of three different generations of superporous hydrogels containing ranitidine. Instead of sodium alginate, chitosan was used as the additional cross-linked hydrophilic polymer that was added in the primary polyacrylamide network [32]. All of these hydrogels had a density lower than 1 g/cm^3^ which enabled their floatation in hydrochloric acid. The incorporation of chitosan in the primary polymeric network (polyacrylamide) enhanced the mechanical stability of the SPHC system, enabling it to withstand a compression force of 3–4 N, while the superporous hydrogel interpenetrating network (SPHIPN) system was elastic enough not to fracture under a 10 N compression force. Drug release from the SPHIPN formulations was characterised by an initial burst release which could be attributed to the API on the surface of the network, followed by sustained release over 24 h. The incorporation of HPMC further sustained the release of the drug. The drug release followed Higuchi kinetics and mainly occurred via diffusion through the polymer matrix [32].

There are some disadvantages to the use of superporous hydrogels for gastric retention purposes. Since ionic polymers are used to a significant extent in the development of these systems, their swelling behaviour can be heavily dependent on gastric pH and, thus, reversible with any pH changes. Furthermore, sometimes they may show poor mechanical strength which can be an issue towards achieving effective gastric retention [10,18].

### 4.6. Osmotic Systems

Osmotic pump technology has been widely used in oral drug delivery. Its applications come with significant advantages. These include zero-order release that is independent of media pH, osmolality and food effects. Additionally, they usually provide good *in vitro*/*in vivo* correlations and a constant drug plasma concentration for the duration of the controlled drug release [173,174]. However, for drugs with a narrow absorption window or drugs absorbed or acting primarily in the upper part of the GIT, the residence of the osmotic system in that area may not be long enough to ensure complete drug release, thus diminishing their bioavailability and therapeutic efficacy [175,176]. Hence, a combination of the osmotic pump technology with a gastroretentive strategy can ensure a prolonged gastric residence time that will allow for a desirable absorption and/or pharmacological action of different drugs. Coreg^®^ CR is a commercial gastroretentive osmotic capsule formulation of carvedilol used to treat high blood pressure and heart failure.

Guan et al. prepared a gastroretentive osmotic capsule formulation with asymmetric membranes containing famotidine as the API [177]. PEO polymers of different molecular weights were tested as suspending and floating agents, NaCl was used as the osmotic agent and cellulose acetate was employed as the coating membrane. Orifices were drilled on either side of the capsule. The results showed that, with regard to floatation, all capsules floated immediately; however, the molecular weight of PEO had a negative effect on the total floating time [177]. The true density of PEO increases with increasing molecular weight [178]. Therefore, this negative effect on total floating time could be due to the fact that at an earlier timepoint the true density of the system was higher than that of the PEO solution formed from the swelling of the polymer in the medium. As a result of the above, due to its low molecular weight and subsequent long floating time of the systems loaded with it, PEO WSR N-80 was selected as the suspending and floating agent. The optimised formulation was selected based on a central composite design and gave a 12-h floatation and drug release *in vitro* that followed zero-order kinetics. Finally, a pharmacokinetic study in Beagle dogs demonstrated a sustained release of famotidine, alongside a superior bioavailability, compared to marketed famotidine tablets [177]. 

Desai et al. employed Quality by Design (QbD) tools to prepare a gastroretentive osmotic system (GROS) tablet formulation containing clopidogrel bisulphate [179]. The formulation consisted of the core tablet which contained NaCl, sodium carboxymethylcellulose, polyvinylpyrrolidone (PVP) and sodium lauryl sulphate (SLS), a cellulose acetate:PEG 4000 coating and the gastroretentive compression coating which contained HPMC K4M, sodium bicarbonate and talc. The optimised tablets conformed to the Quality Target Product Profile (QTPP) specifications as they demonstrated a 12-h zero-order release of up to 90% of the drug, with a negligible floating lag time and a total floating time longer than 12 h. Therefore, the optimised formulation proved to be promising as a gastroretentive osmotic pump delivery system and QbD showed the robust results that can be delivered through its application in the formulation development process [179].

Neumann et al. developed and characterised a novel expandable osmotic system containing furosemide as the API [180]. This system consisted of two main parts. The first was a shield-shaped core oral tablet with the drug release osmotically controlled surrounded by a semi-permeable coating. The tablet was introduced into the second part consisting of swellable polymers, namely HPMC K100M and PEO (POLYOX^®^ WSR-303). The principle of this novel system was to combine the robust release behaviour of an osmotic system with the gastroretentive expandable capabilities of swelling hydrophilic polymers. The system was characterised for its swelling, gastroretentive (using a new mechanical antrum model developed by the group [181]) and *in vitro* release properties, followed by a clinical trial in healthy human subjects. The results demonstrated that the swelling of the system was pH-dependent, while its gastric retention capability could be compromised in reduced media volumes. Both compendial and non-compendial dissolution tests demonstrated a pH-independent, pressure-resistant release of furosemide from the system, however the swellable part was destroyed during the dissolution stress test experiments which could impede the gastric retention of the system *in vivo*. The clinical trial illustrated an effective gastric retention of the system during fed state where the gastric transit time was significantly longer than under fasted conditions. The AUC values recorded during fed state were 10 times higher than the respective values after fasted intake of the system. Therefore, this study demonstrated not only the potential of this novel system for gastroretentive controlled-release applications, but also the value of using biorelevant test methods alongside conventional methods during the development of gastroretentive formulations [180].

Apart from floating and expandable osmotic systems, high-density systems coupled with osmotic technology have been reported. Guan et al. developed a high-density osmotic pump tablet using pharmaceutical iron as a density-increasing, as well as gas-forming, agent. NaCl was the osmotic agent and PEO assumed the roles of suspending agent and release retardant [173]. The swelling of PEO and the hydrogen gas generation resulting from the reaction of pharmaceutical iron with hydrochloric acid were the driving forces for a complete drug release that was controlled over 12 h and followed zero-order kinetics. Furthermore, a gamma scintigraphy study in Beagle dogs demonstrated that the optimised tablets were retained in the animal stomach for 7 h, thus rendering the tablets promising for future applications [173].

### 4.7. Ion-Exchange Resin Systems

Ion-exchange resins are water-insoluble, ionic polymer materials containing two principal parts: a structural portion consisting of a water-insoluble polymer matrix and a functional portion, which is the ion-active group. The ionic groups can be either positively or negatively charged and, therefore, the resins can be either cation- or anion-exchange [182]. Based on the affinity of the ionic groups for soluble counter-ions, the ionic resins are further classified into strong and weak exchangers [183]. Sulphonic acid functional groups tend to be the most common for the strong cation exchangers, while carboxylic acid functional groups are usually present in the weak cation exchangers. For strong anion exchangers, their surface usually contains quaternary ammonium groups, while tertiary amine groups are the most common for the weak anion exchangers [182].

The drugs can be loaded into the ion-exchange resins via two methods, the batch method and the column method [184,185]. During the batch method, a certain amount of resin is immersed into a drug solution and mixing is conducted until equilibrium is reached, while in the column method, a saturated drug solution is passed through a resin-packed column until the effluent concentration and eluent concentration reach an equilibrium [186,187]. Many different factors affect the efficiency and rate of drug loading, including molecular weight and charge intensity of the drug and resin, particle size and cross-linking degree of the resin, as well as nature of the solvent used for the drug solution and mixing conditions. Large drug particle size and a high degree of cross-linking result in slower rates of drug loading, while smaller resin particle size increases the surface area of interaction between the drug and resin, thus potentially accelerating drug loading [186].

Umamaheshwari et al. developed an effervescent gastroretentive cholestyramine-based microcapsule formulation using acetohydroxamic acid as the API [188]. Sodium bicarbonate was bound onto the resin, followed by incorporation of the API, up to a level where the floating of the system was not impeded. After their preparation, the microcapsules were coated with cellulose acetate butyrate (CAB). Drug release from this system was a two-step process, including drug displacement and drug diffusion. The rate of drug release depended on the thickness of the CAB coating and the dissolution medium. Increasing CAB:drug–resin complex ratios led to lower release rates because the increased coating thickness resulted in a longer diffusional path of the drug molecules displaced from the surface of the resin. The drug release from the system was faster in SGF pH 1.2 than phosphate pH 7.4. This can be attributed to the higher affinity of the cholestyramine functional groups towards chloride ions than phosphate ions, due to the initial treatment process of the resin, as well as the size of the ions. Phosphate ions are bulkier than chloride ions which slows their diffusion through the resin particles. The buoyancy lag time of the microcapsules was 2–5 min and they floated for 12 h with a positive effect of increasing CAB:drug–resin complex ratio on the total floating time. The floatation of the system in SGF was achieved through the entrapment of CO_2_ gas into the CAB coating which was generated from the reaction between sodium bicarbonate and hydrochloric acid. The drug release was controlled over more than 8 h. Finally, apart from the floating properties of the microcapsules, their bioadhesion capability was confirmed in a rat stomach. This combination of floating and bioadhesion properties rendered the system promising in achieving robust gastric retention that can help treat *Helicobacter pylori* effectively [188].

The application of ion-exchange systems comes with certain limitations. There are safety issues concerning their ingestion. Furthermore, the amount of resin bound with the drug(s) may be difficult to estimate [10]. Finally, the applications of ion-exchange resins as a controlled-release platform are restricted to drugs with groups that can be charged [186].

### 4.8. Magnetic Systems

**Figure 10:**
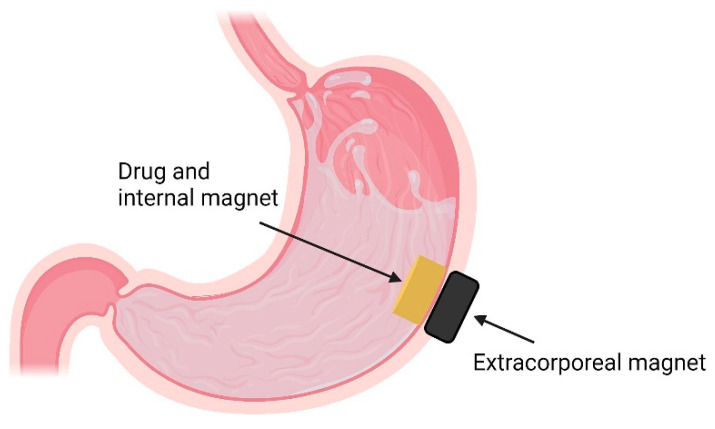
Application of magnetic drug delivery systems for gastric retention purposes. The figure was prepared using BioRender (www.biorender.com) (Accessed 3 August 2021).

In magnetic systems, the distinct feature in the dosage form is a small amount of internal magnet, apart from API and excipients. An extracorporeal magnet is placed over the stomach to control the position of the dosage form (Figure 10) [10]. The gastric retention behaviour of the magnetic systems can be affected by the position and magnetic intensity of the extracorporeal magnet [58].

In the literature, it has been reported that the GRT and bioavailability of drugs are improved using magnetic tablets [34,189]. Gröning et al. tested the gastric retention capability of acyclovir magnetic depot tablets in human volunteers with and without the application of an external magnetic field. The tablets remained in the stomach for a prolonged period in the presence of the extracorporeal magnet and the area under the concentration curve values were significantly increased [35].

Zhou et al. developed gastroretentive adhesive tablets that contained superparamagnetic iron oxide nanoparticles (SPIONPs) which generated microbubbles *in situ* that induced cavitation upon application of ultrasound energy from an external machine [36]. Iron oxide nanoparticles have been reported as a promising nanomedicine in the past, due to their biocompatibility, intrinsic superparamagnetic properties and biodegradability [190,191]. The tablets were prepared by mixing sodium bicarbonate (foaming agent) with HPMC K4M and/or Carbomer 934P which, being swellable hydrophilic polymers, were responsible for the tablet adhesion and/or controlled release of microbubbles from the system. The study results showed that the presence of the foaming agent was crucial in ensuring a stable generation of CO_2_ bubbles *in situ* in an acidic environment, while the presence of an adhesive polymer was necessary for preventing a premature disintegration of the tablets. Furthermore, the combination of HPMC K4M and Carbomer 934P at a ratio of 1:1 was ideal as the adhesive component of the delivery system since, under the application of ultrasound energy, a controlled release of bubbles over 12 h was demonstrated, while the tablets were not completely dissolved. Additionally, increasing the ultrasound intensity applied seemed to significantly increase the release rate of the SPIONPs from the system *in vitro*, whilst accelerating the dissolution of the tablets. Finally, an *ex vivo* study using pig stomach tissue demonstrated an increased penetration and dispersion of the released nanoparticles into the mucosal and muscle layers under ultrasound application, compared to when ultrasound energy was absent. This demonstrated that the release and absorption of nanoparticles can be regulated through the application of external acoustic energy. Furthermore, it was suggested that application of an external magnet could further aid the localisation of the tablets in the stomach and further ensure effective gastric retention [36]. This application could be a promising step towards the development of gastroretentive magnetic nanoparticle-based formulations that will help achieve an effective local and systemic administration of drugs. However, the use of specialised external equipment, such as ultrasound devices and/or external magnets, is essential for desired drug release and gastric retention behaviours of such formulations.

A major challenge associated with the application of magnetic systems is the fact that specific positioning of the magnet can be challenging and could also lead to low patient adherence [58]. Therefore, future research studies on these gastroretentive systems need to focus on their clinical significance.

### 4.9. Combinatory Approaches

Apart from the abovementioned standalone gastroretentive technologies, combinations have been reported. Combinatory approaches can help tackle the disadvantages of each of the different technologies combined. Additionally, they can help minimise the variability of GRT and may be less affected by the physiological conditions of the stomach, such as those during fasted and fed state, thus ensuring robust gastric retention [10]. These combinations could be floating and expandable systems, mucoadhesive and expandable systems and floating and mucoadhesive systems. The first two combinations have indicated to have higher safety and efficacy for clinical applications [192].

#### 4.9.1. Floating and Expandable Systems

This category includes GRDDS that are designed to float and expand or swell. These formulations need to possess four characteristics. To begin with, the initial dosage form must be small enough for swallowing. Once the dosage form reaches the stomach after co-administration with water, it should float and remain buoyant to avoid a premature evacuation from the stomach. Then, as it comes in contact with the gastric fluids, it should rapidly expand to a certain size that will prevent its passage through the pyloric sphincter. Finally, when there is no need for the formulation to be gastroretentive anymore, it can be reduced to a size that will allow gastric emptying [87,193,194]. Highly swellable polymers, such as HPMC K4M, K15M and K100M, PEO and chitosan can be used to achieve a desirable swelling index for the formulations [2,105,195].

Chen et al. developed losartan GRDDS tablets containing a mixture of hydroxyethylcellulose and chitosan as swelling polymers, while sodium bicarbonate was used as a gas-generating agent [2]. A decrease in the viscosity of chitosan resulted in better swelling behaviour of the formulation, probably due to more rapid penetration of the medium in the tablet which promoted the swelling of hydroxyethylcellulose. Additionally, sodium bicarbonate seemed to have a negative effect on the swelling of chitosan, probably due to the neutralisation of the medium pH around the tablets which potentially resulted in hindrance of the polymer swelling. Therefore, the optimum polymer:sodium bicarbonate ratio was crucial in obtaining the preferred behaviour of GRDDS. The optimised tablets contained an equivalent ratio of the two polymers and were able to control the release of losartan over 16 h *in vitro*, whilst demonstrating a good swelling and floating behaviour of the system The drug release potentially followed the Case II diffusion model kinetics, meaning that it followed the rate of swelling of the hydrogel matrix which led to a constant release [2].

**Figure 11:**
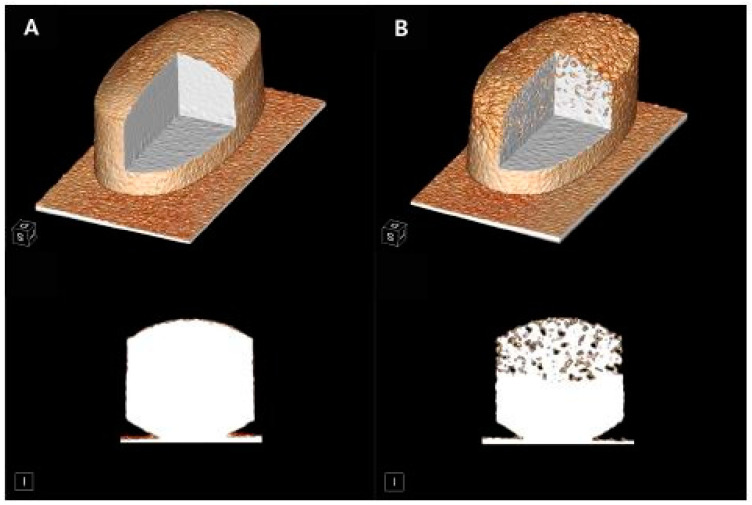
Images from 3D X-ray microcomputed tomography showing the non-porous tablet structure before sublimation (**A**) and the highly porous structure of the gastroretentive layer of the tablets after sublimation (**B**). Reprinted from International Journal of Pharmaceutics Vol 572, Hwang et al., Swellable and porous bilayer tablet for gastroretentive drug delivery: Preparation and *in vitro*-*in vivo* evaluation, 118783 (Pages 1–13), Copyright 2019, with permission from Elsevier.

Hwang et al. developed swellable, highly porous bilayer tablets containing ranitidine [105]. The gastroretentive layer consisted of a swellable polymer and had a highly porous structure, due to the removal of volatile materials during a sublimation process step. Different hydrophilic polymers and sublimating agents were tested for their properties to choose the most suitable materials for the gastroretentive layer. Eventually, PEO was selected as the optimal polymer, due to its persistent swelling over 12 h and its ability to ensure a high tablet wet strength, as well as maintaining a high tablet tensile strength even after sublimation. Between camphor and menthol, camphor was chosen as the optimal sublimating agent, due to its significantly higher sublimation rate and its much smaller negative effect on tablet tensile strength, compared to menthol. Additionally, HPMC K4M was incorporated into the formulation to retard the release of ranitidine. X-ray microcomputed tomography of the tablets demonstrated a highly porous internal tablet structure where the pores were evenly distributed (Figure 11), thus ensuring a sufficiently low density for a robust floatation and suggesting that buoyancy could be maintained even after a slight surface erosion of the gastroretentive layer *in vitro* or *in vivo*. The tablets were able to retard the release of ranitidine over 12 h *in vitro* with the release significantly affected by the amount of HPMC K4M, the percolation threshold of which was determined to be between 11.48 and 21.69% v/v. Finally, an *in vivo* study in Beagle dogs demonstrated the ability of the gastroretentive layer to ensure gastric retention of tablets, especially under fed conditions, thus rendering the developed system a promising technology for a stomach-specific delivery of drugs [105].

Lin et al. developed formulations where PEO and Kollidon^®^ SR were used as release retardants and gastroretentive agents. Single-layer tablets were initially developed, however, despite their floatability, their hardness was too low for practical use, along with a very low drug release extent, therefore they were not forwarded to *in vivo* studies [87]. Caplets of a similar formulation that were developed did not float, while capsules that contained the same amount of API and excipients showed good swelling and floating behaviour, along with a release of the API over 24 h [87].

#### 4.9.2. Mucoadhesive and Expandable Systems

Different studies in the literature have demonstrated the promising capability of mucoadhesive and expandable systems in achieving effective gastric retention for the release of drugs in the upper part of the GIT.

Zentner et al. developed chitosan:PVP hydrogels at a weight ratio of 2:1. The brittle solid mixture swelled extensively (110–115 fold) and rapidly (within less than 3 h) when in contact with acidic water (pH 2.0) [196]. This system could therefore be promising as an expandable system with mucoadhesive properties that are probably linked to hydrogen and electrostatic interactions between chitosan and the mucus [150,197]. Furthermore, Su et al. developed polyionic complex hydrogels of chitosan with ring-opened (ro) PVP containing alendronate in an attempt to provide a system with improved stability, mucoadhesive and swelling properties that would ensure robust gastric retention in the upper part of the GIT, alongside a controlled release of the drug [47]. Lyophilised powders consisting of different complexes of chitosan of different molecular weights and roPVP prepared via heat application and the addition of sodium hydroxide were compressed into tablets. Mucoadhesive measurements demonstrated a synergistic enhancement of mucoadhesion for chitosan/roPVP complex hydrogels. The viscosity and mucoadhesion force values increased significantly with increasing percentage of chitosan and the complex hydrogels were able to interact more strongly with mucin, compared to chitosan, irrespective of the molecular weight of chitosan. In terms of *in vitro* drug release, the chitosan/roPVP complex hydrogels of all different chitosan molecular weights provided a 24-h release of alendronate. The release rate increased with increasing swelling ratio of the polymers, and therefore it was suggested that an increase in the gel volume rendered the drug diffusion pathway less hindered, thus increasing the release rate. So, the high-molecular-weight chitosan/roPVP complex hydrogel tablets demonstrated the highest release rate. Finally, an *in vivo* pharmacokinetic study in rabbits indicated the ability of the high-molecular-weight chitosan/roPVP complex polymer to be successfully retained in the stomach and release alendronate slowly over time, providing increased AUC, half-life and bioavailability values, along with a lower C_max_ value, compared to a quarter of a Fosamax^®^ tablet. Therefore, the combination of strong swelling and mucoadhesive properties of the chitosan/roPVP complex polymer-based tablets resulted in a new promising formulation that can reduce the risk of alendronate toxicity, along with improving its efficacy [47].

**Figure 12:**
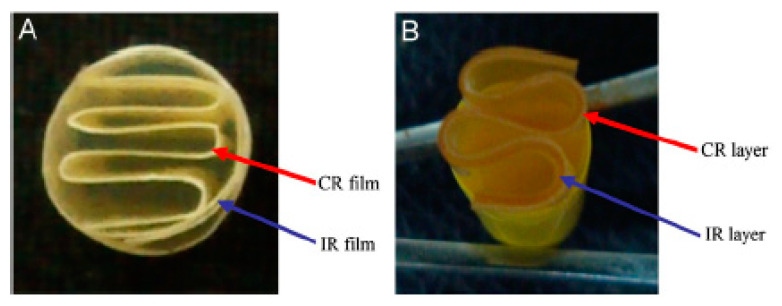
The film folding patterns in case I (**A**) bilayer capsules where the furosemide immediate-release film was rolled around the zig-zag-folded controlled-release film and case II (**B**) bilayer capsule formulations where both films were folded in a zig-zag manner. Reprinted from Acta Pharmaceutica Sinica B Vol 2, Darandale et al., Design of a gastroretentive mucoadhesive dosage form of furosemide for controlled release, Pages 509–517, Copyright 2012, published under a Creative Commons Attribution 3.0 License (https://creativecommons.org/licenses/by-nc-nd/3.0/, accessed on 2 August 2021). No changes were made to the original figure presented as Figure 1 in the original research paper.

Apart from beads, another potentially efficient bioadhesion and floatation combination technique involves the use of unfolding and swelling polymeric films. Darandale et al. developed a furosemide bilayer capsule formulation consisting of an immediate-release polymeric film and a controlled-release mucoadhesive folded film [198]. Two different formulations were prepared. In case I, the controlled-release film was folded inside the capsule in a zig-zag manner and the immediate-release film was rolled over it, while in case II both films were folded inside the capsule in a zig-zag manner (Figure 12). It was realised that in case II the zig-zag geometry helped the controlled-release film unfold properly in acidic media. The mucoadhesion and controlled release of furosemide from the formulation was achieved mainly due to the presence of Carbopol^®^ 971P, the carboxylic acid groups of which formed hydrogen bonds with the mucus layer of Wistar rat stomach mucosa. Additionally, the presence of HPMC E4M contributed to the swelling of the film and the controlled release of the API which followed Fickian diffusion. Based on its effective unfolding, mucoadhesion and controlled-release properties, the formulation could be most promising *in vivo* [198].

#### 4.9.3. Floating and Mucoadhesive Systems

Various *in vitro* and *in vivo* studies have been carried out on the development of mucoadhesive floating drug delivery systems designed to improve gastric residence of drugs through a combination of mucoadhesion and floating mechanisms [2,29,198,199,200]. In a study, a hollow-bioadhesive microsphere formulation containing glyceryl monooleate as a bioadhesive polymer and psoralen as the model drug was developed [201]. These microspheres showed strong mucoadhesive properties, good buoyancy and a sustained drug release *in vitro* and in Sprague Dawley rats where an extension of the half-life and a reduction in the elimination rate of the drug were recorded, compared to a psoralen suspension [201].

Karemore et al. focused on the development of a cilnidipine effervescent floating gastroretentive tablet formulation that contained gellan gum as the bioadhesive polymer, as well as HPMC K4M and sodium bicarbonate as excipients that can aid in floatation and controlled drug release [202]. The amounts of these three components had a statistically significant effect on the floating lag time, total floating time, mucoadhesion and *in vitro* drug release properties of the tablets. An increase in the amount of these components significantly reduced the floating lag time and prolonged the floatation of tablets. This was due to the increased swelling of the polymers, alongside the entrapment of CO_2_ bubbles generated from the reaction of sodium bicarbonate with hydrochloric acid [202]. Furthermore, sodium bicarbonate acted as a source of sodium ions which promoted the *in situ* gelation of GG through the formation of a cross-linked, three-dimensional network [203,204,205,206]. Regarding the mucoadhesive capability of the tablets, increasing amounts of GG demonstrated an increasing mucoadhesive strength of the tablets onto goat stomach mucosa, due to increased water uptake and swelling and higher availability of adhesive polymer sites that could interact with the mucin molecules. The presence of HPMC K4M significantly enhanced the mucoadhesive strength of the tablets, probably due to a physical entanglement of the two polymers, as well as non-covalent bonding. The drug release was prolonged for 12 h *in vitro* with HPMC K4M and GG being the significant factors affecting it. The drug release was found to be governed by non-Fickian diffusion which includes a combination of diffusion and erosion mechanisms. Finally, the tablets demonstrated promising gastric retention and drug release results in human volunteers, indicated by a prolonged half-life and reduced elimination rate of cilnidipine, compared to conventional tablets. These findings further demonstrated the potential of the cilnidipine gastroretentive tablet application [202].

Abd El-Aziz et al. developed alfuzosin gastroretentive sponges based on chitosan and HPMC. The sponges proved to be highly porous with density values significantly below 1.004 g/cm^3^ which accounted for their immediate floatation [207]. Furthermore, the mucoadhesive force was measured to be higher for the chitosan-based sponges, potentially due to electrostatic interactions between the positively charged amino groups of chitosan and negatively charged sialic acid and mucin. Additionally, the polymer grade and concentration impacted the release of alfuzosin from the system since higher polymer concentration could lead to the formation of a more rigid gel barrier that could reduce water ingress into the matrix, thus reducing the drug release rate. The mechanism of drug release from the optimised sponges was found to be non-Fickian (anomalous) diffusion through a combination of drug diffusion and erosion of the polymeric matrix. Finally, the *in vivo* gastroretentive potential of the optimised sponges was confirmed in healthy male volunteers [207].

Bera et al. prepared olive oil-entrapped alginate beads containing risperidone, coated with an ionotropically cross-linked alginate-sterculia gum blend gel. The authors believed that this combination would achieve gastric retention through a combination of floatation and mucoadhesion [200]. The uncoated beads were optimised in terms of their drug entrapment efficiency and *in vitro* drug release. Then, the optimised beads were coated with the blend gel and the coated beads were assessed in terms of their physical properties, buoyancy, *in vitro* drug release and *ex vivo* mucoadhesion in the goat stomach mucosa. The results demonstrated that drug entrapment efficiency was significantly dependent on the polymer-to-drug and oil-to-water ratios in the uncoated beads, while drug release rate increased with increasing polymer-to-drug ratio and decreasing oil-to-water ratio. After coating with the ionotropically cross-linked alginate-sterculia gum blend gel, the release of risperidone was successfully retarded over 8 h in SGF, and it followed a Fickian diffusion-based mechanism. The beads were able to float rapidly due to their low bulk density [200]. The oil entrapment into the beads played a vital role in their buoyancy, due to the formation of multiple pockets within the polymer matrix [208]. The floating lag time decreased with increasing oil content. Furthermore, the swelling and expansion of the polymer matrix further contributed to the buoyancy of the beads via an additional reduction in their density. Finally, the *ex vivo* study demonstrated an effective adherence of the coated beads onto goat stomach mucosa in an acidic environment, thus rendering the formulation promising as a gastroretentive drug delivery system for risperidone [200]. In another study, Dey et al. developed alginate beads as floating and mucoadhesive GRDDS using sunflower oil as a low-density component to achieve immediate and prolonged buoyancy of the beads, alongside a controlled drug release through the creation of oil pockets in the polymer matrix [199].

Darbasizadeh et al. reported the development and characterisation of ranitidine tripolyphosphate (TPP)-cross-linked chitosan/PEO electrospun nanofibrous mats [12]. The cross-linking process was crucial in ensuring the mechanical stability of the nanofibres and their integrity in aqueous environment given the degradability of the constituent polymers. Furthermore, cross-linking was crucial in ensuring robust mucoadhesive properties of the nanofibres since the cross-linked fibres interacted more strongly and for a significantly longer time with goat stomach mucosa *ex vivo*, compared to uncross-linked ones. Additionally, the prepared cross-linked nanofibres floated immediately in SGF, due to their high surface area and porosity, and their buoyancy was maintained over more than 24 h. In terms of *in vitro* drug release, the cross-linked nanofibres demonstrated a reduced burst release, compared to the non-cross-linked ones, and a significantly prolonged release of ranitidine up to 24 h. This was proposed to be due to ionic interactions between tripolyphosphate groups of TPP and ammonium groups of chitosan that may have resulted in reduced mobility and swelling degree of the polymer chains [12]. Therefore, this non-effervescent nanofibre formulation could be promising as a controlled-release gastroretentive delivery system, due to its versatility and its floating and mucoadhesive properties. The potential of cross-linked chitosan and PEO combinations in nanofibre preparation was also reported by Abd El Haby et al. who, apart from *in vitro* and *ex vivo* characterisation, conducted an *in vivo* study in rats for the prepared nanofibres. The results demonstrated a robust gastroprotective activity of nizatidine that was significantly more potent when formulated in cross-linked nanofibres, compared to drug solution and uncross-linked nanofibres [209].

## 5. Future Considerations

GRDDS-based controlled-release applications are promising in the oral delivery of drugs that can either act locally towards treating conditions of the upper part of the GIT or act systematically, but may have certain limitations, such as pH-dependent solubility or stability, a narrow window of absorption and/or short half-life. Many gastroretentive controlled-release products are already in the market, with most based on a single-system approach.

All different categories of GRDDS have their limitations. However, the development of systems with combinatory approaches, such as the ones reported in the present review, are promising in achieving a robust behaviour in terms of effective gastric retention and controlled release of different drugs. Furthermore, potential safety issues related to some of the single-approach systems can be addressed through combinatory approaches. The floating and expandable combinatory systems could potentially be the most promising as a technology for the future development of GRDDS since it can offer prolonged GRT via two robust mechanisms of gastric retention without the risk of gastric or bowel obstruction and gastropathy that can be conferred through the use of the expandable systems as an only approach.

*In vivo* monitoring of the gastric residence of GRDDS is crucial in assessing the gastric retention capability of the gastroretentive formulations. For this purpose, different imaging methods have been employed. These include scintigraphy, X-rays, magnetic resonance imaging (MRI), magnetic moment imaging, gastroscopy and ultrasonography. Each of these imaging techniques has its advantages and limitations [210]. However, these techniques have not been used extensively in the literature to assess the ability of the GRDDS to remain in the stomach for the desired length of time. It is typically considered adequate to simply record the pharmacokinetic profile of drugs after oral administration of the dosage forms and make inferences on the gastric retention capability of the formulations based on the profile parameters. However, *in vivo* monitoring of the GRDDS is a very important part of the formulation development and should be reported, whenever the appropriate equipment is available.

## 6. Conclusions

The application of GRDDS coupled with controlled-release strategies is a promising approach to improve the therapeutic efficacy and dosing regimen of drugs with solubility, stability, half-life and/or absorption limitations that can treat a wide range of diseases either locally, such as *Helicobacter pylori* infections, or systemically, such as type 2 diabetes or Parkinson’s disease. The anatomy and physiology of the stomach under different states (fasted, fed) and individual patient conditions need to be taken into account during formulation development and clinical use, alongside any critical attributes of the system, based on the chosen approach. There are many different approaches for the development of GRDDS and a few commercially available pharmaceutical products are based on these technologies. However, single-system approaches are accompanied by limitations that could impede a robust formulation behaviour. Combinations of the different technologies could help overcome the limitations of individual approaches, thus rendering these combinatory systems most promising in achieving effective gastric retention and controlled release of a wide range of suitable drugs. The *in vivo* imaging of gastroretentive formulations can further assist in assessing the robustness of their gastric residence.

## Figures and Tables

**Table 1:** Gastroretentive products currently under clinical trials or available commercially.

Delivery System	Brand Name	Active Pharmaceutical Ingredient	Phase	Manufacturing Company
Hydrodynamically Balanced	Madopar^®^	Levodopa and Benserazide	Commercial	Intec Pharma (Israel)
	Valrelease^®^	Diazepam	Commercial	Roche (UK)
Non-effervescent floating tablet	Glucophage^®^ XR	Metformin	Commercial	Merck KGaA (Germany)
Effervescent floating	Cifran^®^	Ciprofloxacin	Commercial	Ranbaxy (India)
Raft-forming	Liquid Gaviscon^®^	Sodium bicarbonate and Calcium carbonate	Commercial	Reckitt Benckiser Healthcare (UK) Ltd.
	Gaviscon^®^ Tablets	Sodium bicarbonate and Calcium carbonate	Commercial	Reckitt Benckiser Healthcare (UK) Ltd.
	Topalkan^®^	Aluminium and Magnesium	Commercial	Pierre Fabre Medicament (France)
Mucoadhesive/Bioadhesive	Xifaxan^®^	Rifaximin	Commercial	Lupin (India)
Expandable	Accordion Pill^®^ Levodopa/Carbidopa	Levodopa and Carbidopa	Phase III Clinical Trials	Intec Pharma (Israel)
	Requip^®^ XL (Geomatrix^®^ technology)	Ropinirole	Commercial	GlaxoSmithKline (UK)
	Glumetza^®^ (Acuform^®^ technology)	Metformin	Commercial	Salix (USA)
	Nucynta^®^ ER (Acuform^®^ technology)	Tapentadol	Commercial	Depomed (USA)
	Gralise^®^ (Acuform^®^ technology)	Gabapentin	Commercial	Depomed (USA)
	Janumet^®^ XR	Sitagliptin and metformin	Commercial	Merck Sharp & Dohme (USA)
	LYN-005 (Long-acting Pill technology)	Risperidone	Phase II Clinical Trials	Lyndra^®^ Therapeutics (USA)
Osmotic	Coreg^®^ CR	Carvedilol Phosphate	Commercial	GlaxoSmithKline (UK)

## Data Availability

Not applicable.
